# Supplementary material for: Abrupt structural transition in exotic molybdenum isotopes unveils an isospin-symmetric island of inversion
Source: Nat Commun. 2025 Nov 27;16:10631. doi: 10.1038/s41467-025-65621-2 (PMC12661037; doi:10.1038/s41467-025-65621-2)
Supplement: Supplementary file 1 — Supplementary Information [file 41467_2025_65621_MOESM1_ESM.pdf]

# Supplementary Information

# Abrupt Structural Transition in Exotic Molybdenum Isotopes unveils an Isospin-Symmetric Island of Inversion

J. Ha[1,2,3,4], F. Recchia[2,3*], S. M. Lenzi[2,3], H. Iwasaki[5,6], D. D. Dao[7], F. Nowacki[7], A. Revel[5,6], P. Aguilera[2,3], G. de Angelis[8], J. Ash[5,6], D. Bazin[5,6], M. A. Bentley[9], S. Biswas[5], S. Carollo[2,3], M. L. Cortes[8], R. Elder[5,6], R. Escudeiro[2,3,10], P. Farris[5,6], A. Gade[5,6], T. Ginter[5], M. Grinder[5,6], J. Li[5], D. R. Napoli[8], S. Noji[5], J. Pereira[5], S. Pigliapoco[2,3], A. Pompermaier[2], A. Poves[11], K. Rezynkina[2,3], A. Sanchez[5,6], R. Wadsworth[9] and D. Weisshaar[5]

[1]Center for Exotic Nuclear Studies, Institute for Basic Science, 34126 Daejeon, Republic of Korea.
[2]Dipartimento di Fisica, Università degli Studi di Padova, I-35131 Padova, Italy.
[3]Sezione di Padova, Istituto Nazionale di Fisica Nucleare, I-35131 Padova, Italy.
[4]Instituut voor Kern- en Stralingsfysica, KU Leuven, B-3001 Leuven, Belgium.
[5]Facility for Rare Isotope Beams, Michigan State University, MI 48824 East Lansing, USA.
[6]Department of Physics and Astronomy, Michigan State University, MI 48824 East Lansing, USA.
[7]Université de Strasbourg, CNRS, IPHC UMR7178, F-67000 Strasbourg, France.
[8]Laboratori Nazionali di Legnaro, Istituto Nazionale di Fisica Nucleare, I-35020 Legnaro, Italy.
[9]School of Physics, Engineering and Technology, University of York, YO10 5DD York, United Kingdom.
[10]Instituto de Fisica, Universidade de São Paulo, 05315-970 São Paulo, Brazil.
[11]Departamento de Física Teórica and IFT UAM-CSIC, Universidad Autónoma de Madrid, E-28049 Madrid, Spain.
*Corresponding author. E-mail: francesco.recchia@pd.infn.it

# Supplementary Figures

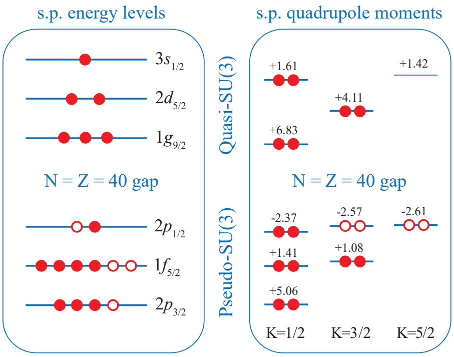


**Supplementary Figure 1 Configuration in $^{84}$Mo.** Quasi- and pseudo-SU(3) plot are displayed for the 8p-8h configuration in $^{84}$Mo. Only the picture for one type of nucleons is shown as the configuration is the same for protons and neutrons. Left: spherical single-particle levels in the ZBM3 model space, particles as full circles, holes as empty circles. Right: single-particle quadrupole moments characterized by the projection of the total angular momentum on the symmetry axis, $K$.

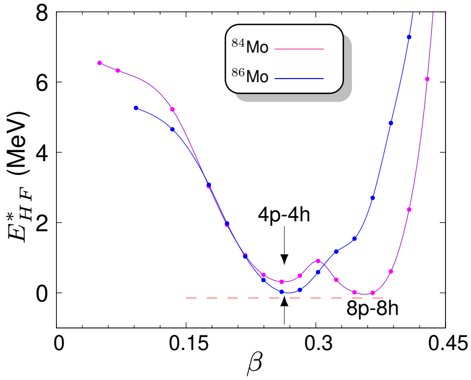


**Supplementary Figure 2 Deformation curve versus $\beta$.** Deformation curve representing the Hartree-Fock energy variation with respect to $\beta$ where $\gamma$ is fixed to the HF minimum one and $E^*_{HF}$ measures the energy difference at the point $\beta$ with respect to the HF minimum. To guide the eye, the dashed red line and arrows indicate its position and particle-hole structure.

# Supplementary Tables

**Supplementary Table 1 Probability amplitudes of configurations in $^{84,86}$Mo.** Extraction of normalized probability amplitudes ($P_\alpha^{(J)}(\beta,\gamma)$) of several leading configurations $(\beta,\gamma)$ in the ground-state of $^{84,86}$Mo, as depicted in Fig. 4, together with their associated n$p$-n$h$ excitation structures. $E^*_{HF}$ measures the Hartree-Fock energy difference of a given $(\beta,\gamma)$ configuration with respect to the HF minimum (shown in red).

| nucleus | $\beta$ | $\gamma$ ($deg$) | $P_\alpha^{(J)}(\beta,\gamma)$ (%) | n$p$-n$h$ | $E^*_{HF}$ (MeV) |
|---|---|---|---|---|---|
| | 0.37 | 22.2 | 11.61 | **8$p$-8$h$** | 0.0 |
| | 0.35 | 19.1 | 11.93 | 8$p$-8$h$ | 0.03 |
| | 0.32 | 12.9 | 5.93 | 8$p$-8$h$ | 0.82 |
| | 0.37 | 16.0 | 5.84 | 8$p$-8$h$ | 0.38 |
| | 0.32 | 19.1 | 8.25 | 8$p$-8$h$ | 0.37 |
| $^{84}$Mo | 0.39 | 22.2 | 8.29 | 8$p$-8$h$ | 0.61 |
| | 0.37 | 9.8 | 4.02 | 8$p$-8$h$ | 1.46 |
| | 0.39 | 16.0 | 4.42 | 8$p$-8$h$ | 1.24 |
| | 0.35 | 9.8 | 5.90 | 8$p$-8$h$ | 1.04 |
| | 0.50 | 6.7 | 1.58 | 8$p$-8$h$ | 1.51 |
| | 0.27 | 27.3 | 6.15 | 4$p$-4$h$ | 0.0 |
| | 0.32 | 22.2 | 1.53 | 6$p$-6$h$ | 1.07 |
| | 0.28 | 25.3 | 8.07 | 4$p$-4$h$ | 0.09 |
| | 0.24 | 31.5 | 3.23 | 4$p$-4$h$ | 0.34 |
| | 0.32 | 9.8 | 0.38 | 6$p$-6$h$ | 2.84 |
| $^{86}$Mo | 0.26 | 43.9 | 2.35 | 4$p$-4$h$ | 0.74 |
| | 0.30 | 22.2 | 5.40 | 4$p$-4$h$ | 0.66 |
| | 0.37 | 37.7 | 0.18 | 6$p$-6$h$ | 4.73 |
| | 0.13 | 28.4 | 0.14 | 2$p$-2$h$ | 4.63 |
| | 0.18 | 12.9 | 0.14 | 2$p$-2$h$ | 3.71 |
| | 0.24 | 22.2 | 3.46 | 4$p$-4$h$ | 0.60 |
| | 0.28 | 59.4 | 0.44 | 4$p$-4$h$ | 1.95 |
